# Aqueous humor renin, angiotensin I, and angiotensin II activity in primary open-angle glaucoma

**DOI:** 10.5935/0004-2749.20200052

**Published:** 2020

**Authors:** Valéria Batista Boreck Seki, Guilherme Rabelo de Souza, Andre Messias, Dulce Elena Casarini, Jayter Silva de Paula

**Affiliations:** 1 Department of Ophthalmology, Otorhinolaryngology and Head and Neck Surgery, Faculdade de Medicina de Ribeirão Preto, Universidade de São Paulo, Ribeirão Preto, SP, Brazil; 2 Nephrology Division, Department of Medicine, Universidade Federal de São Paulo, São Paulo, SP, Brazil

**Keywords:** Glaucoma, open-angle, Cataract, Aqueous humor, Renin-angiotensin system, Glaucoma de ângulo aberto, Catarata, Humor aquoso, Sistema renina-angiotensina

## Abstract

**Purpose:**

The renin-angiotensin system is involved in the pathogenesis of retinal ischemic conditions and glaucoma. Our objective was to evaluate the renin, angiotensinconverting enzyme 1, and angiotensin-converting enzyme 2 activities in aqueous humor and blood samples of patients with and without primary open-angle glaucoma.

**Methods:**

We analyzed samples from 56 participants who underwent ocular surgeries. The patients were divided into two groups: patients with cataract alone (n=28) and patients with cataract and primary open-angle glaucoma (n=28). Venous blood (2 ml) and aqueous humor (150 µl, *via* paracentesis) samples were collected during phacoemulsification (cataract only) or glaucoma surgery (cataract and primary open-angle glaucoma). The serum and aqueous humor renin, angiotensin-converting enzyme 1, and angiotensin-converting enzyme 2 activities of all patients were evaluated by fluorimetric assays, and results were analyzed by using multivariate regression analysis.

**Results:**

Both the aqueous humor renin activity and renin activity aqueous humor/serum ratio were significantly lower in patients with cataract and primary open-angle glaucoma than in patients with cataract only [(mean ± SE): 0.018 ± 0.006 ng/ml/h vs 0.045 ± 0.009 ng/ml/h, p<0.001; 0.05 ± 0.02 vs 0.13 ± 0.05, p=0.025]. Multivariate analyses showed a significant relationship between lower aqueous humor renin activity and primary open-angle glaucoma [coefficient (±SE): -0.029 ± 0.013, p=0.026].

**Conclusions:**

Our results showed that patients with primary open-angle glaucoma had lower aqueous humor renin activity. As timolol eye drops were used by most of the primary open-angle glaucoma patients, we propose that a large sample of washed-out patients should be studied in the future to discriminate the involvement of b-blocker treatment in the aqueous humor renin activity.

## Abstract (pt)

**Objetivo:**

O sistema renina-angiotensina está envol vido na patogênese das condições isquêmicas retinianas e no glaucoma. Nosso objetivo foi avaliar a atividade da renina, enzima conversora de angiotensina 1 e 2 no humor aquoso, e amostras de sangue de pacientes com e sem glaucoma primário de ângulo aberto.

**Métodos:**

Foram analisadas amostras de 56 participantes submetidos à cirurgia ocular. Os pacientes foram divididos em dois grupos: pacientes com catarata apenas (n=28), e pacientes com catarata e glaucoma primário de ângulo aberto (n=28). Amostras de sangue venoso (2ml) e humor aquoso (150 µl, via paracentese) foram coletadas durante a facoemulsificação (apenas catarata) ou cirurgia de glaucoma (catarata e glaucoma primário de ângulo aberto). As atividades sérica do humor aquoso de renina, enzima conversora de angiotensina 1 e enzima conversora de angiotensina 2 de todos os pacientes foram avaliadas por ensaios fluorimétricos, e os resultados foram analisados por regressão multivariada.

**Resultados:**

Tanto a atividade da renina no humor aquoso quanto à razão humor aquoso/soro da atividade da renina foram significativamente menores nos pacientes com catarata e glaucoma primário de ângulo aberto do que em pacientes com catarata apenas [(média ± DP): 0,018 ± 0,006 ng/ml/h vs 0,045 ± 0,009 ng/ml/h; p<0,001 e 0,05 ± 0,02 vs 0,13 ± 0,05; p=0,025]. Análises multivariadas mostraram uma releção significativa entre menor atividade de renina no humor aquoso e glaucoma primário de ângulo aberto [coeficiente (±erro padrão): -0,029 ± 0,013; p=0,026].

**Conclusões:**

Como a maioria dos pacientes com glaucoma primário de ângulo aberto usavam o colírio de timolol, estudos futuros envolvendo um maior número de pacientes e retirada prévia do tratamento são necessários para se discriminar o envolvimento do uso de betabloqueadores na atividade da renina no humor aquoso.

## INTROD UCTION

Visual loss prevention is the main goal of glaucoma treatment, which involves the reduction of intraocular pressure (IOP)^(1)^. Therapeutic approaches that directly target tissues involved in changes in aqueous humor (AH) outflow may lead to improvements in long-term IOP control in the future.

Several results of ischemia have been associated with damage to AH outflow tissues in primary open-angle glaucoma (POAG)^(2)^. Cellular and extracellular matrix changes in the trabecular meshwork and Schlemm’s canal result in increased IOP due to worsening outflow resistance under pathological conditions^(2-5)^. In glaucoma, the trabecular meshwork may shift from “thin and distensible” to a thicker and stiffer condition. Findings related to such structural modifications include a local increase in levels of transforming growth factor-b-2, a potent factor related to protein deposition and increasing stiffness in the extracellular matrix^(3,4)^.

The renin-angiotensin system (RAS) is involved in systemic hemodynamics and has been associated with fibrosis in several tissues, including the eye^(6)^. In this system, angiotensin II (Ang II) is produced by angiotensin-converting enzyme type 1 (ACE1) and is related to several tissue modifications through its effect on angiotensin receptor type 1, such as fibrotic responses to strain force^(7)^. Renin is the first enzyme in this system, responsible for the production of angiotensin I, and is formed by the cleavage of (pro)renin^(8)^.

In glaucoma, an early insight regarding the influence of RAS in IOP was described in 1988: Constad et al. showed that lower IOP resulted from the use of an ACE1 inhibitor in glaucoma patients^(9)^. Recently, losartan (an angiotensin receptor type 1 inhibitor) was proposed for use in glaucoma treatment^(10)^. Moreover, stimulation of ACE2 with diminazene aceturate was shown to cause production of angiotensin-(1-7) [Ang-(1-7)] and IOP reduction in experimental rats^(11)^.

Moreover, the RAS is known to be involved in pathological events related to ischemia and oxidative stress in many tissues, including the eye; however, key points of this process have not been fully elucidated in glaucoma^(12)^. Because the modulation of RAS elements has shown IOP-lowering effects in glaucoma, we hypothesized that the RAS may be involved in pathological changes observed in the anterior segment of patients with glaucoma. To test this hypothesis, this preliminary study was performed to evaluate the renin, ACE1, and ACE2 levels in the AH and blood samples of patients with and without POAG.

## METHODS

### Participants

Participants were prospectively selected from the Glaucoma Outpatient Service of the Ribeirão Preto Clinical Hospital (Ribeirão Preto Medical School, University of São Paulo, Brazil) during the period from January 2017 to August 2018. The study protocol adhered to the tenets of the Declaration of Helsinki and was approved by the local institutional ethics committee. Informed consent was obtained from all participants.

Based on the results of a previous study regarding renin activity measured in the vitreous body of patients with diabetic retinopathy, compared with controls, we used a standard deviation of 0.20 ng/ml/h, a significance level of 0.05, a sample power of 85%, and a projected loss of 20% of patients; we determined that at least 40 subjects (20 participants per group) were needed to detect a mean difference between groups of 0.22 ng/ml/h^(13)^.

We included patients with cataract who were scheduled for phacoemulsification, as well as patients with both cataract and POAG who were scheduled for phacoemulsification with or without trabeculectomy. All patients exhibited best-corrected visual acuity worse than 20/40 and had a spherical equivalent within ±6 diopters. The diagnosis of POAG (with or without cataract) was previously confirmed by medical records indicative of glaucomatous optic neuropathy (vertical cup to disc ratio ≥0.7 or asymmetry > 0.2, neuroretinal rim thinning or notching, and localized or diffuse retinal nerve fiber layer defect), open angles, at least two Goldmann tonometry readings >21 mmHg, and an abnormal standard automated perimetry 24-2 visual field (SITA-STANDARD; Humphrey Visual Field Analyzer 750, Carl Zeiss, Dublin, CA, USA), as previously defined^(14)^.

### Renin, ECA1, and ECA2 activities

A single-blinded collection of both AH and blood samples was performed during the surgical procedures. After routine anesthesia, the surgeon tapped the anterior chamber using a BD Ultra-Fine^TM^ 29G 0.5-inch disposable syringe needle in the peripheral temporal region of clear cornea. One hundred fifty microliters of AH were slowly aspirated and immediately placed into sterile cryotubes for freezing in liquid nitrogen. The same volume of balanced saline solution or surgical viscoelastic solution was then injected to restore the anterior chamber through the same puncture hole, and the procedure was continued in accordance with the protocol previously determined by the surgeon. After completion of the surgery, 2 mL of peripheral blood was collected from each participant, centrifuged to separate plasma, and frozen for posterior analysis.

The levels of active forms of renin in the AH and plasma were analyzed using an enzymatic activity assay, as previously described^(15)^. Renin activity was determined using the spectrofluorometer F-200 (Infinite Model; Tecan, Switzerland) with the substrate Abz-DRVYIHPFHLLVYSQ-EDDnp (10 um). Aliquots of samples were preincubated in buffer (50 mM tris, pH 7.5, containing a protease inhibitor cocktail) at 37°C for 8 min. For the renin inhibition test, the aliskiren specific inhibitor was added. The fluorescent substrate was then added and diluted in assay buffer. Readings were collected every 2 min for 60 min (16) (excitation: 320 nm; emission: 420 nm) at 37°C. Calculations were based on the standard curve OMNI, discounting the zero time of reaction and the values of the test with inhibition of each timepoint.

The ACE1 catalytic activity was determined by fluorimetry, as described previously^(16,17)^. Briefly, aliquots of samples (10 µL each) were incubated with Z-Phe-His-Leu (ZPhe-HL; 1 mM; 200 µL) in 100 mM sodium borohydride, pH 8.3, 300 mM NaCl, and 0.1 mM ZnSO_4_. The enzymatic reaction was terminated by the addition of NaOH (0.28 N; 1.5 mL), and samples were incubated with *o*-phthaldialdehyde (20 mg/mL methanol; 100 µL; 10 min). The fluorescence reaction was terminated by the addition of HCl (3 N; 200 µL). The dipeptide His-Leu (L-HL) thus released was measured fluorometrically (λ: 365 nm; λem: 495 nm) using the spectrofluorometer F-200 (Infinite Model; Tecan).

The ACE2 catalytic activity was also determined by fluorimetry. Samples were homogenized in buffer (75 mM Tris, pH 6.5, 1 M NaCl, and 0.5 µM ZnCl_2_), with a protease inhibitor cocktail (Complete Mini EDTA-free, Roche) and 10 µM captopril. ACE2 activity was determined in spectrofluorometer (Tecan, Switzerland), using the substrate MCA-APK-Dnp (excitation: 320 nm; emission: 420 nm). Buffer and samples were incubated at 37°C; substrate was then added, and sample readings were collected for 90 min. Arbitrary units were registered, calculations were based on a fluorescence standard curve OMNI (OMNIMMP^®^ fluorogenic control); timepoint 0 was used as an internal blank. The protocol was based on a previously described method, with modifications^(18)^.

### Statistical analyses

Data were analyzed using descriptive and inferential statistics, including analysis of contingency tables for frequencies (Stata version 14.2; StataCorp LLC, College Station, TX, USA). Comparisons between groups were performed regarding enzymatic activities in the AH and serum, as well as the AH-serum enzymatic ratio, using the Mann-Whitney U test (significance level of p<0.05). Linear regression with continuous and categorical predictors was applied to evaluate associations between each type of enzymatic activity in AH and blood samples and the following factors: age, sex, use of systemic antihypertensive drugs, and ocular and systemic diagnoses (significance level of p<0.05).

## RESULTS

Fifty-six of the 95 AH and blood samples collected (95 participants; one eye/subject) could be used for enzymatic analyses. Of the 56 participants analyzed [mean (±standard deviation) age: 71.9 ± 7.3 years; 26 men (46.4%)], 28 had cataract only, whereas 28 had cataract + POAG. The clinical findings of all participants are presented in table 1.

**Table 1:** Clinical and demographic characteristics of the 56 participants for whom aqueous humor and blood samples were analyzed.

Characteristic	Cataract (n=28)	POAG (n=28)
Age (years)^*^	71.9 ± 5.6	71.9 ± 9.1
Sex (male/female)	13:15	13:15
Use of systemic antihypertensive drugs		
ACE inhibitor (yes/no)	7/21	3/25
Angiotensin 11 receptor antagonist (yes/no)	9/19	8/20
β-blockers (yes/no)	13/15+	5/23†
Systemic diseases (n)		
None	0	1
Systemic hypertension	17	19
Diabetes mellitus	0	3
Combined^**^	6	4
Other	5	1

POAG= primary open-angle glaucoma; ACE= angiotensin-converting enzyme; other= other systemic disease;

* = mean ± standard deviation;

** = systemic arterial hypertension and diabetes mellitus present in the same patient.

† = p<0.05 (Fisher’s exact test).

**Figure 1:**
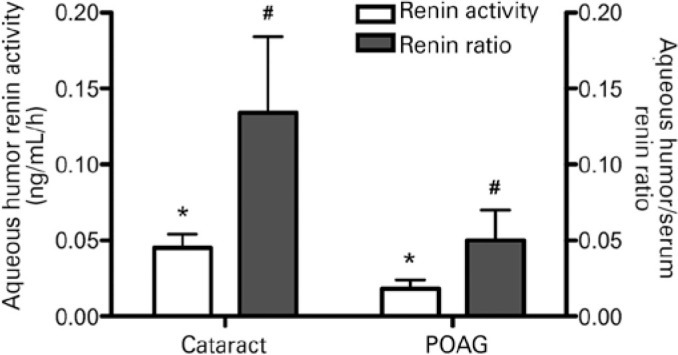
Aqueous humor (AH) renin enzymatic activities and AH-serum renin activity ratios of the 56 participants. Cataract group serves as control for comparison with patients with primary open-angle glaucoma (POAG).

Both AH renin activity and renin activity AH-serum ratio were significantly lower in patients with cataract and POAG than in patients with cataract only [(mean ± SE): 0.018 ± 0.006 ng/ml/h versus 0.045 ± 0.009 ng/ml/h, p<0.001; 0.05 ± 0.02 versus 0.13 ± 0.05, p=0.025] (Figure 1). A complete description of all enzymatic findings is presented in table 2. No additional differences in enzymatic activity were observed based on comparisons regarding age, sex, use of systemic ACE inhibitors and angiotensin II receptor antagonists, or past ocular surgeries. Of note, more patients in the group with ca taract only were using systemic _b_-blockers [13/28 (cataract only) versus 5/28 (cataract and POAG), p=0.0437], and only patients with POAG were using topical _b_-blockers [27/28 (cataract and POAG) versus 0/28 (cataract only), p<0.0001].

**Table 2:** Enzymatic activity in aqueous humor and blood samples of the 56 participants, separated by group.

Samples (Mean ± standard error)	Cataract (n=28)	POAG	
		(n=28)	P-value
Renin (Aqueous Humor)	0.045 ± 0.011	0.018 ± 0.006	<0.001
Renin (Blood)	0.668 ± 0.251	0.824 ± 0.497	0.994
ACE1 (Aqueous Humor)	0.945 ± 0.095	1.024 ±0.108	0.799
ACE1 (Blood)	70.64 ± 5.68	87.05 ± 6.53	0.309
ACE2 (Aqueous Humor)	0.024 ± 0.003	0.029 ± 0.004	0.862
ACE2 (Blood)	0.167 ± 0.019	0.188 ± 0.030	0.425

POAG= primary open-angle glaucoma; ACE= angiotensin-converting enzyme.

*: significant at p<0.05 between comparable data - Mann-Whitney U test.

Linear regression to adjust for multiple comparisons revealed a significant relationship between lower AH renin activity and POAG [coefficient (±SE): -0.029 ± 0.013, p=0.026].

## DISCUSSION

We investigated the activity of selected RAS components in blood samples and AH of patients with cataract and compared them with samples from patients with cataract and POAG. We hypothesized that RAS would be involved in oxidative stress events that contribute to the pathophysiology of ischemic ocular diseases and POAG. Following multivariate analysis of samples obtained from 56 patients, we found significantly lower AH renin activity in patients with cataract and POAG than in patients with cataract only.

To the best of our knowledge, this is the first observation of a remarkable reduction in AH renin activity in patients with POAG. RAS components may have distinct roles in inflammation and neovascularization, and some studies have detected ocular renin modulation of retinal ischemic conditions and glaucoma^(8-11,13,19-22)^.

Renin inhibitors have been tested in ischemic retina^(21,22)^ and have been shown to reduce IOP^(23,24)^. Based on preexisting descriptions of increased ocular renin in glaucoma, as well as reduction of IOP following treatment with renin inhibitors, our observation of significantly lower AH renin activity in patients with POAG may be deemed contradictory. To the best of our knowledge, few conditions could cause the reduction of AH renin activity. Neither a local increase in the consumption of renin nor a preferential shift toward binding to the (pro)renin receptor and consequent activation of this alternative RAS pathway have been studied in eyes of patients with glaucoma. Nonetheless, the use of _b_-blockers has been associated with a systemic reduction in renin activity, potentially due to reduced renin release^(25)^ through a mechanism involving cAMP^(26)^. Patients with glaucoma are frequently treated with topical _b_-blockers (e.g., timolol maleate 0.5%) and might exhibit lower local renin relea se to the AH. Although the use of systemic _b_-blockers was more frequently observed in patients with cataract only in the present study, its use limited the analysis of an association with renin activity, considering that 27/28 (96.4%) of the POAG patients were using timolol eye drops, and 28/28 (100%) of the cataract patients did not use them. In this scenario, the assigned groups (cataract and POAG/ cataract only) and the treatment (using/not using systemic or topical _b_-blockers) were confounding factors, regardless of multivariate analysis.

In conclusion, our investigation of the enzymatic activity of selective RAS components showed that patients with cataract and POAG had lower AH renin activity measurements than patients with cataract only. Because most patients with POAG in this study were using timolol maleate eye drops, further studies are needed, using a larger sample of patients with a previous washout period, to confirm our findings and verify whether topical b-blocker treatment is involved in reducing the release of renin to the AH.
